# Temporal Trends and Climatic Factors Associated with Bacterial Enteric Diseases in Vietnam, 1991–2001

**DOI:** 10.1289/ehp.9658

**Published:** 2007-10-16

**Authors:** Louise A. Kelly-Hope, Wladimir J. Alonso, Vu Dinh Thiem, Do Gia Canh, Dang Duc Anh, Hyejon Lee, Mark A. Miller

**Affiliations:** 1 Division of International Epidemiology and Population Studies, Fogarty International Center, National Institutes of Health, Department of Health and Human Services, Bethesda, Maryland, USA; 2 National Institute of Hygiene and Epidemiology, Hanoi, Vietnam; 3 International Vaccine Institute, SNU Research Park, Seoul, Korea

**Keywords:** cholera, climate, dysentery, enteric disease, epidemiology, outbreaks, seasonality, shigellosis, typhoid fever, Vietnam

## Abstract

**Objective:**

In Vietnam, shigellosis/dysentery, typhoid fever, and cholera are important enteric diseases. To better understand their epidemiology, we determined temporal trends, seasonal patterns, and climatic factors associated with high risk periods in eight regions across Vietnam.

**Methods:**

We quantified monthly cases and incidence rates (IR) for each region from national surveillance data (1991–2001). High- and low-disease periods were defined from the highest and lowest IRs (1 SD above and below the mean) and from outbreaks from positive outliers (4 SDs higher in 1 month or 2 SDs higher in ≥ 2 consecutive months). We used general linear models to compare precipitation, temperature, and humidity between high- and low-risk periods.

**Results:**

Shigellosis/dysentery was widespread and increased 2.5 times during the study period, with the highest average IRs found between June and August (2.1/100,000–26.2/100,000). Typhoid fever was endemic in the Mekong River Delta and emerged in the Northwest in the mid-1990s, with peaks between April and August (0.38–8.6). Cholera was mostly epidemic along the central coast between May and November (0.07–2.7), and then decreased dramatically nationwide from 1997 onward. Significant climate differences were found only between high- and low-disease periods. We were able to define 4 shigellosis/dysentery, 14 typhoid fever, and 8 cholera outbreaks, with minimal geotemporal overlap and no significant climatic associations.

**Conclusions:**

In Vietnam, bacterial enteric diseases have distinct temporal trends and seasonal patterns. Climate plays a role in defining high- and low-disease periods, but it does not appear to be an important factor influencing outbreaks.

In Vietnam, shigellosis (bacillary dysentery), typhoid fever, and cholera are enteric diseases of significant public health concern (DeRoeck et al. 2005). They are primarily caused by the bacterial pathogens *Shigella* spp., *Salmonella typhi,* and *Vibrio cholerae,* respectively, and transmission occurs through fecal contamination of food or water or by person-to-person contact (Bhan et al. 2005; Crump et al. 2004; Kindhauser 2003; Kotloff et al. 1999; Lanata et al. 2002). Infection rates and outbreaks are highest where the standards of living, water supply, and human behaviors related to personal hygiene and food preparation are poor. The distribution and ecologic determinants of shigellosis/dysentery, typhoid fever, and cholera have recently been described from surveillance data in Vietnam (Kelly-Hope et al. 2007). The data show that each disease varies in magnitude and has a distinct spatial pattern, which appears to be driven by a combination of human and environmental factors, including poverty, water sources, and climate.

Many infectious diseases, including shigellosis/dysentery, typhoid fever, and cholera, are influenced by climate. Specifically, climate plays an important role in the transmission process and can influence spatial and seasonal distributions, as well as interannual variability and long-term trends [Burke et al. 2001; Kovats et al. 2003; World Health Organization (WHO) 2004]. Although climate is one aspect of the complex epidemiology of these enteric diseases, it can help to define high-risk periods. Few studies conducted in Asia have described the temporal patterns and outbreaks of shigellosis/dysentery and typhoid fever, and no study has specifically examined the impact of climate on these diseases. In general, cholera has been studied more widely, and formal and informal listings of outbreaks and putative risk factors are available from various sources (Griffith et al. 2006; Kelly-Hope et al. 2007; WHO 2003, 2005, 2006). Studies have shown associations of *V. cholerae* with climate, including rainfall, flooding, water temperature and depth, sea surface temperatures, and the El Niño Southern Oscillation (ENSO) (Huq et al. 2005; Koelle et al. 2005b; Lipp et al. 2002; Lobitz et al. 2000; Pascual et al. 2000; Rodo et al. 2002).

In Vietnam, monthly shigellosis/dysentery, typhoid fever, and cholera surveillance data have been collated for 1991–2001. We used these national data to determine the long-term temporal trends and seasonal patterns of shigellosis/dysentery, typhoid fever, and cholera in eight geographic regions of Vietnam, and to examine climatic factors associated with high-risk periods.

## Methods

### Study location

**Figure 1:**
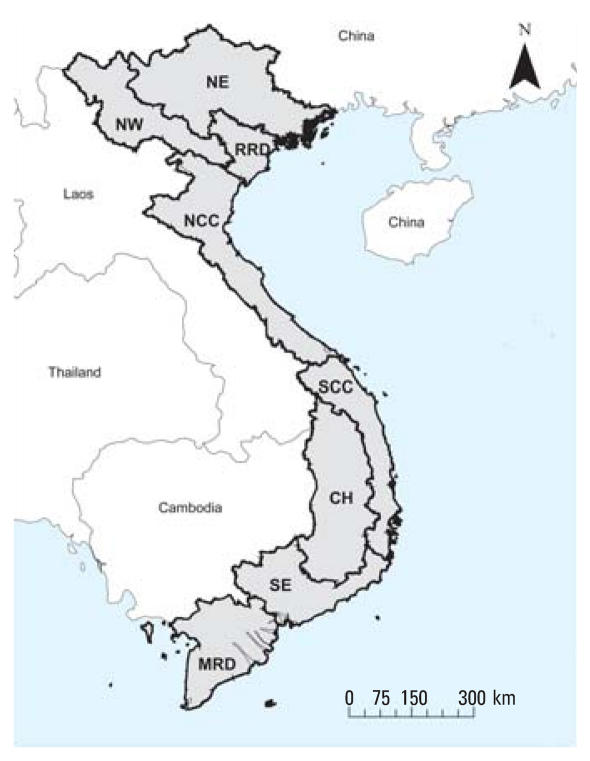
Vietnam and its eight regions: Northeast [NE; 8,524,800 (average population for 1996)], Northwest (NW; 2,112,900), Red River Delta (RRD; 16,331,800), North Central Coast (NCC; 9,696,100), South Central Coast (SCC; 6,287,300), Southeast (SE; 10,947,300), Central Highlands (CH; 3,563,000), and Mekong River Delta (MRD; 15,693,500).

Vietnam is a narrow, densely populated country in southeastern Asia bordering China, Laos, and Cambodia (General Statistics Office of Vietnam 2005). It has approximately 85 million people living in an area of 330,000 km^2^, with > 3,000 km of coastline. In the south the climate is tropical, whereas in the north, the two main seasons are a warm, wet summer and a cool, humid winter. The terrain is diverse with low, flat deltas in the south and north; highlands in the center; and hilly mountains in the northwestern region. Vietnam experiences occasional typhoons with extensive flooding, especially in the southern Mekong River Delta. Vietnam currently is divided into 64 provinces and eight agro-ecologic regions (Figure 1): Northeast, Northwest, Red River Delta, North Central Coast, South Central Coast, Central Highlands, Southeast, and Mekong River Delta. We used the eight geographic regions as the basis of our temporal and climatic analyses.

### Disease data

We obtained data on shigellosis/dysentery, typhoid fever, and cholera for each province in Vietnam from 1991 to 2001 from the Epidemiology Department, National Institute of Hygiene and Epidemiology (Hanoi), and from a central database collated by the International Vaccine Institute (Korea). Data were primarily (> 90%) based on treated episodes, which are routinely collected by district health centers as part of the surveillance system of the Vietnam Ministry of Health; these episodes were supplemented with cases reported in the published scientific literature and unpublished national health reports. Thus, the database comprised a combination of cases that were diagnosed clinically and confirmed by serology and stool culture. Provincial data were pooled to provide estimates for each of the eight study regions.

### Temporal trends and seasonal patterns

To determine long-term temporal trends and seasonal patterns of shigellosis/dysentery, typhoid fever, and cholera, we quantified the monthly number of cases and average incidence rates (IRs) per 100,000 population for each region. Population data for 1995–2001 were obtained from the General Statistics Office of Vietnam (2005), and population estimates for 1991–1994 were extrapolated from the fitted cubic spline of the known years (Eubank 1999) in order to obtain regional population estimates and crude IRs for each study year.

To identify distinct seasonal variations, we detrended (with a fourth-degree polynomial) and log-transformed monthly IRs in each region for each disease, and defined “high” and “low” disease periods based on the months with the highest and lowest rates (months with values at least 1 SD above and below the mean, respectively). Outbreak periods were detected similarly, but we defined them empirically as the positive outliers that were 4 SDs higher in 1 month or 2 SDs higher in ≥ 2 consecutive months from the modeled Fourier function of the time series (Bloomfield 2000; Pollock 1999), which was performed on each time series, accounting for disease seasonality.

### Climate data and analysis

Monthly climatic data were obtained from worldwide climate maps generated by the interpolation of data from ground-based meteorologic stations with a monthly temporal resolution and 0.5° (latitude) by 0.5° (longitude) spatial resolution (Mitchell and Jones 2005). The climatic variables used were precipitation; average daily minimum, maximum, and mean temperatures; vapor pressure; and number of wet days. Monthly climate data during 1991–2001 were extracted from the pixels containing the centroid of each province and clustered according to the eight regional divisions of Vietnam. To calculate climatic averages for the eight regions, we used the climatic values for each province weighted by its respective population (to account for the proportional relevance of the diseases of each province within the regions, so the climatology of places where few people live would, in fact, account proportionally less in the regional analyses than places with a large demographic concentration).

To explore climatic factors associated with high-risk times, we examined differences between high- and low-disease periods and outbreak and non-outbreak periods. First, we used a general linear model to test significant differences between high- and low-disease periods with time lags from 0 to 2 months. Because multiple tests were conducted (four climatic variables tested at three time lags of 0, 1, and 2 months, thus yielding 12 tests for each disease at each region), significance levels were adjusted with the Bonferroni correction (Sokol and Rohlf 1995); we considered *p*-values < 0.05/12 significant.

Second, we compared climate data corresponding to the outbreak period in each region with climate data for the same months in previous years when outbreaks did not occur (i.e., the non-outbreak period), with time lags from 0 to 2 months. We used general linear models with the climate variables as dependent variables, outbreak presence as a fixed factor, and region as a random factor.

All analyses were performed using Microsoft Excel (Microsoft Corporation, Redmond, WA, USA), ArcGIS 9.1 (ESRI, Redlands, CA, USA), and MATLAB software (The MathWorks, Inc., Natick, MA, USA).

## Results

### Temporal trends and seasonal patterns

**Figure 2:**
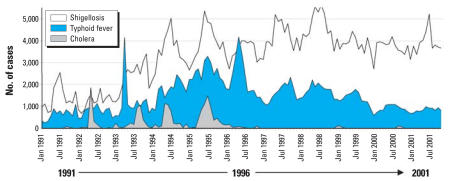
The monthly number of shigellosis/dysentery, typhoid fever, and cholera cases reported in Vietnam during 1991–2001.

The monthly numbers of shigellosis/dysentery, typhoid fever, and cholera cases reported in Vietnam during 1991–2001 are shown in Figure 2. Shigellosis/dysentery was the most prevalent disease and increased approximately 2.5 times during the study period, with 16,976 cases (annual IR of 25.3 per 100,000) reported in 1991 compared with 46,292 cases (IR, 58.8) in 2001. The annual number of typhoid fever cases was similar at the beginning (7,592 cases; IR, 11.3) and end (9,614 cases; IR, 12.2) of the study period; however, there was a 3-fold increase during 1994 to 1997, with an average of 24,553 cases (IR, 33.8) reported annually. Overall, there were fewer cholera cases, which appeared episodically during 1991–1996, with four main peaks in May 1992 (1,851 cases; IR, 2.7), August–September 1993 (943–1,054 cases; IR, 1.4–1.5), May 1994 (1,127 cases; IR, 1.6), and June–July 1995 (1,097–1,492 cases; IR, 1.5–2.1). From January 1997 onward, the number of cholera cases reported nationwide decreased significantly, with only two minor peaks reported in January–February 1999 (188 cases; IR, 0.25) and September–October 2000 (166 cases; IR, 0.21).

**Figure 3:**
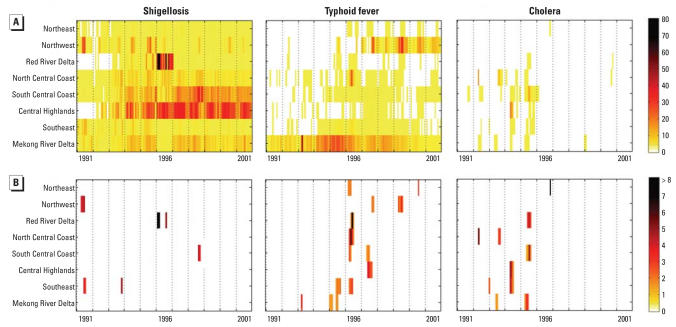
Monthly incidence rates per 100,000 population and outbreaks of shigellosis/dysentery, typhoid fever, and cholera in eight regions of Vietnam. (*A*) Incidence rates. (*B*) Outbreaks. Dotted vertical lines define years, and individual bands indicate values for months; geographic regions are sorted by latitude.Outbreaks are displayed as SD above the modeled Fourier function.

Figure 3A shows the monthly IRs of shigellosis/dysentery, typhoid fever, and cholera for each region during 1991–2001. This figure highlights the widespread incidence of shigellosis/dysentery and its increase in the Central Highlands and the South Central Coast, the endemicity of typhoid fever in the Mekong River Delta and its emergence in the Northwest region, and the significant decline of cholera nationwide.

**Figure 4:**
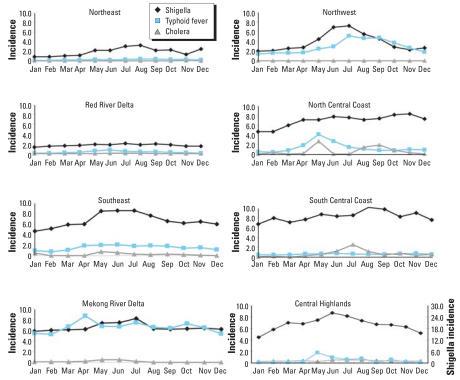
Average monthly shigellosis/dysentery, typhoid fever, and cholera incidence rates per 100,000 population in eight regions of Vietnam. Note the different scale for shigellosis in the Central Highlands.

Overall, we found distinct seasonal variations in each region, as shown by the average monthly IRs in Figure 4. Shigellosis/dysentery rates peaked in the northern regions of the country (Northeast, Northwest, Red River Delta, North Central Coast) between June and August (IR range, 2.1–7.8), and in the southern regions (South Central Coast, Central Highlands, Southeast, Mekong River Delta) between May and July (IR range, 8.2–26.2); the highest monthly IR occurred in the Central Highlands in June (IR, 26.2). Typhoid fever rates peaked in the northern regions between May and September (IR range, 0.38–5.2) and in the southern regions between April and July (IR range, 0.43–8.6); the highest monthly IRs occurred in the Northwest in July (IR, 5.2) and the Mekong River Delta in April (IR, 8.6). Cholera rates peaked in the northern regions between May and November (IR range, 0.07–2.7) and in the southern regions between May and July (IR range, 0.51–2.6). No cholera cases were reported in the Northwest, whereas the highest monthly IRs occurred in the North Central Coast in May (IR, 2.7) and in the South Central Coast in July (IR, 2.6).

In total, 26 enteric outbreaks were identified—4 shigellosis/dysentery, 14 typhoid fever, and 8 cholera—during 1991–2001 (Figure 3B). Apart from typhoid and cholera in the Mekong River Delta in June 1995, no disease outbreak coincided temporally with any other disease outbreak in any region. However, typhoid outbreaks in the Northeast, Red River Delta, North Central Coast, South Central Coast, and Southeast regions in 1996 overlapped temporally, with outbreak months ranging from March to July. Overall, outbreaks occurred most commonly in the months of May, June, and July, followed by April, August, and September. No outbreaks occurred in December, and only one to three outbreaks occurred in October–March.

### Climate associations

The climatic measures during high- and low-disease periods at 0-month lag are shown in Table 1. The data highlight that, in most regions, conditions were warmer, wetter, and more humid in high-disease periods than in low-disease periods. Overall, we found significant differences in precipitation and the number of wet days between the high and low periods. For shigellosis/dysentery and cholera, precipitation was significantly different (*F*_1,11_ = 14.7, *p* = 0.002, *r^2^*_adj_ = 47.7%; and *F*_1,10_ = 15.7, *p* = 0.002, *r^2^*_adj_ = 53.1%, respectively), as was the number of wet days (*F*_1,11_ = 18, *p* = 0.001, *r^2^*_adj_ = 53.2%; and *F*_1,10_= 14.4, *p* = 0.003, *r^2^*_adj_ = 50.7%, respectively) at the 0-month time lag. Similarly, for typhoid fever, precipitation was significantly different at the 0-month time lag (*F*_1,11_ = 40.1, *p* < 0.001, *r^2^*_adj_ = 72.3%), as was the number of wet days at the 0- and 1-month time lags (*F*_1,11_ = 24.8, *p* < 0.001, *r^2^*_adj_ = 61.4%; and *F*_1,11_ = 28.1, *p* < 0.001, *r^2^*_adj_ = 64.3%, respectively). No significant climatic differences were found at the 2-month time lag for any of the diseases, even when tests were not Bonferroni adjusted.

In our climate analyses we found no significant differences in the climatic conditions between the months during or preceding each outbreak period compared with non-outbreak periods in previous years. The data in Table 2 highlight the range of climate conditions under which enteric outbreaks occurred. Overall, precipitation ranged from 37 to 311 mm; for the majority (> 80%) of the outbreaks, > 100 mm was recorded. All mean temperatures were > 21.9°C (majority > 25°C); the number of wet days ranged from 4.9 to 20.3 (majority > 11); and most outbreaks occurred in months with an average vapor pressure > 26 hPa.

## Discussion

This is the first time that temporal patterns of endemic and epidemic shigellosis/dysentery, typhoid fever, and cholera have been defined concurrently on such a large scale. In the present study we used surveillance data to highlight the different magnitudes and epidemiologic patterns of each disease in Vietnam during 1991–2001, and we offer some insight into the role of climate. Notwithstanding the inherent limitations associated with surveillance data, this large data set is probably the most comprehensive available in any developing country, and provides the basis for more specific and well-defined hypotheses in relation to climate and disease.

Overall, we found that the incidence of shigellosis/dysentery was widespread and increased significantly during the study period, especially in the Central Highlands and South Central Coast. The reported dysentery could have been caused by other pathogens such as *Campylobacter* or *Escherichia coli* (Isenbarger et al. 2001, 2002; Ngan et al. 1992); however, *Shigella* spp. are the most common cause of dysentery, with four distinct species able to exist in a range of ecologic niches (Kotloff et al. 1999). Also, new variants have potentially emerged in Vietnam (Isenbarger et al. 2001). In addition, the increase in shigellosis/dysentery may be related to widespread antibiotic resistance (Anh et al. 2001; Isenbarger et al. 2001, 2002; Nguyen et al. 2005; Vinh et al. 2000) and the fact that no vaccines or alternative treatments are available. Thus, shigellosis is potentially one of the most important enteric pathogens in Vietnam.

We found typhoid fever concentrated in three regions of the country, each with differing temporal patterns. In the Mekong River Delta the disease was endemic and rates were among the highest in the country, which supports previous studies (Lin et al. 2000; Luxemburger et al. 2001; Nguyen et al. 1993). In the central region of Vietnam, especially the North Central Coast, South Central Coast, and Southeast, a substantial increase occurred between 1995 and 1998, which may account for the high number of cases reported nationwide and the series of outbreaks we identified during this period. In the Northwestern region, typhoid fever first appeared in 1996–1997 and remained endemic thereafter (Tran et al. 2005). The reason for its emergence and persistence in this remote rural region is unclear. It is possible that ENSO, which resulted in extremely hot conditions across the country in 1997–1998, somehow enhanced the transmission of *Salmonella typhi* in this region or endemicity is related to new border openings.

In contrast, cholera decreased dramatically from 1997 onward, and many regions reported no further cases after years of epidemic and endemic activity (Dalsgaard et al. 1999). This sudden widespread reduction in cholera may be attributable to several factors, including interannual variability, immunity, economic development, and improvements and interventions in hygiene and sanitation. The initial decline probably reflects the episodic nature of cholera. Other studies have also shown that interannual variability is common and is affected by climate and events such as the ENSO, as well as by levels of immunity within populations (Koelle et al. 2005a, 2005b; Lipp et al. 2002; Lobitz et al. 2000; Pascual et al. 2000; Rodo et al. 2002).

However, the fact that cholera numbers remained low from 1997 to 2001 may be related to the introduction of a new locally produced vaccine in 1997 (Trach et al. 1997; Vu et al. 2003) instead of ENSO influence, given that in 1996 there were already virtually no reported cases of cholera (with the exception of the outbreak in the Northeast region). Public health campaigns and > 5 million doses of the cholera vaccine targeting both *V. cholerae* 01 and 0139 pathogens have since been distributed primarily to epidemic-prone regions via the national vaccine program, thus influencing the epidemiology of cholera (Thiem et al. 2006). It is impossible to know which factor is most responsible for this decline and almost disappearance of cholera in Vietnam, but this success is undoubtedly due to a combination of public health interventions, including water and sanitation improvements, vaccine delivery to high-risk populations, and changes in public awareness, as well as cyclical population immunity.

Identifying peak periods of disease helps to focus local interventions. We were able to better define the seasonality of each disease and found that, on average, the highest IRs of shigellosis/dysentery occurred between May and August; of typhoid fever between April and September; and of cholera between May and November. For all diseases, the highest monthly IRs occurred earlier (April/May to July) in the southern regions than in the northern regions (May/June to November) of the country, which may be indicative of the different climatic patterns of the north and south. In particular, the tropical conditions of the south may help local health authorities implement timely interventions because peak periods of disease coincided with the onset of the wet season.

Distinct climatic differences were evident between the high- and low-disease periods, with hotter, wetter, and more humid conditions associated with an increased incidence of disease. Climatic associations, however, were not strong, and we found significant differences mainly when we compared the high-and low-disease periods (0-month lags) and not the months leading up to (2-month lag) each specific period. This may be because high and low periods occurred during more extreme climate conditions (i.e., wet and dry seasons) and because climate conditions outside these parameters are more variable and not specific enough to dramatically increase or decrease disease transmission.

The overall weak association with climate could also be related to the quality of surveillance data, which are inevitably flawed because of underreporting, misdiagnosis, and misclassification. In Vietnam, adequate diagnostic facilities are not universally available, and detection can be difficult and may be biased to those individuals with severe symptoms or better access to health centers (Dalsgaard et al. 1999; Hong et al. 2003). Further, other factors such as poor socioeconomic conditions play a role (Fewtrell et al. 2005: Kelly-Hope et al. 2007) and are also likely to be as important, if not more important, than climate. This theory is supported by our analysis of outbreaks, which found no significant climatic differences in the same months between years with outbreaks and years without outbreaks.

Using a robust method, we were able to define statistically 4 outbreaks of shigellosis/ dysentery, 14 of typhoid fever, and 8 of cholera. We found little or no overlap between outbreaks of the three diseases within each region, which suggests that a combination of different factors triggered each event in each region, and that competition may have occurred between these enteric microbes for available hosts (Rabbani and Greenough 1999). Comparisons of climatic factors between outbreak and non-outbreak periods indicated that no specific or unusual climate conditions preceded any outbreak. However, most outbreaks occurred within certain periods and climatic parameters, with May, June, and July being the most common outbreak months, followed by April, August, and September.

We acknowledge that climate is only one aspect of a multitude of complex interactions that cause disease. Although the role of climate is limited, we believe that climate factors help define high- and low-risk periods and potentially provide some clues into the ecology and epidemiology of these enteric diseases. It is reasonable to expect that the different pathogens, as well as humans, respond to seasonal changes in the environment and that some conditions are more favorable than others for disease transmission.

## Correction

In the original manuscript published online, “vapor pressure” and “number of wet days” were incorrectly labeled in the “Results” and in Tables 1 and 2 because of an error in the climate data set. These have been corrected here.

## Figures and Tables

**Table 1:** Differences in climatic factors during high- and low-disease periods in Vietnam during 1991–2001.

	Shigellosis				Typhoid fever				Cholera			
Region	PREC	TEMP	WET	VAP	PREC	TEMP	WET	VAP	PREC	TEMP	WET	VAP
Northeast												
High	236.7	26.5	12.8	25.8	172.7	26.5	12.1	25.1	149.7	25.0	10.7	23.2
Low	61.5	20.0	10.5	17.5	97.5	21.4	10.6	19.2	109.8	22.8	10.1	20.6
Northwest												
High	272.0	26.0	17.0	25.2	226.3	24.7	16.5	23.5	NR	NR	NR	NR
Low	60.2	19.7	10.7	16.5	118.7	22.11	12.8	19.7	NR	NR	NR	NR
Red River Delta												
High	117.7	23.6	11.5	22.3	228.5	28.0	13.0	28.4	297.4	29.1	16.6	30.9
Low	111.7	23.7	10.9	22.4	38.0	19.1	8.2	16.2	141.4	23.8	11.0	22.7
North Central Coast												
High	187.4	25.4	12.1	24.5	170.3	25.4	13.6	24.4	245.7	26.1	15.2	26.1
Low	113.7	22.0	10.5	20.2	85.7	21.7	11.1	19.8	168.7	23.5	11.5	22.3
South Central Coast												
High	158.5	25.3	13.8	25.4	199.5	26.5	13.8	27.2	185.8	26.3	19.1	26.1
Low	187.6	24.3	12.2	24.6	161.3	24.4	12.0	24.5	134.2	24.2	11.6	24.2
Central Highlands												
High	168.6	25.7	14.8	25.7	185.6	26.3	15.7	26.2	233.8	25.6	19.3	26.6
Low	92.8	23.5	8.5	23.1	133.6	23.8	9.8	23.7	122.2	24.1	11.3	23.8
Southeast												
High	202.1	27.9	14.5	28.6	221.8	27.6	15.7	28.8	224.6	27.1	15.3	28.1
Low	59.1	26.4	7.2	24.9	66.3	26.3	8.0	24.7	136.7	26.9	11.5	26.7
Mekong River Delta												
High	204.6	28.7	14.2	30.2	209.0	28.2	13.9	28.8	186.0	29.0	14.1	30.1
Low	145.4	27.7	11.6	27.5	102.9	27.2	9.5	11.2	159.1	27.5	12.6	27.6

NR, not reported; PREC, precipitation (mm); TEMP, mean temperature (°C); VAP, vapor pressure (hPa); WET, wet days (number in month).

**Table 2:** Region, year, month, and average climate measures for shigellosis/dysentery, typhoid fever, and cholera outbreaks.

			Climate measures			
Region	Year/month	Disease	PREC	TEMP	WET	VAP
Northeast	1996/Mar–May	Typhoid	116.3	24.1	10.7	21.9
	1996/Nov	Cholera	46.9	21.9	4.9	18.3
	2000/Aug	Typhoid	193.2	28.7	11.4	28.8
Northwest	1991/May–Jul	Shigellosis	249.4	26.6	16.0	26.0
	1997/Sep–Oct	Typhoid	132.7	24.7	11.7	23.0
	1999/May–Aug	Typhoid	253.2	26.7	18.6	26.1
Red River Delta	1995/Jun–Aug	Cholera	311.4	28.7	16.2	30.4
	1996/May–Jul	Typhoid	213.0	28.9	12.5	29.3
North Central Coast	1996/Apr–Jul	Typhoid	177.0	26.2	15.1	25.9
South Central Coast	1995/May–Aug	Cholera	142.0	27.0	19.3	26.4
	1996/Apr–May	Typhoid	71.2	25.1	10.5	25.6
	1997/May–Jul	Typhoid	121.5	28.5	15.2	28.2
	1998/Aug–Sep	Shigellosis	239.2	27.3	20.3	27.1
Central Highlands	1994/May–Jul	Cholera	135.7	26.0	18.7	26.8
	1997/Jun–Sep	Typhoid	230.6	28.8	19.8	29.4
Southeast	1991/Jul–Aug	Shigellosis	258.5	27.8	16.9	29.8
	1993/Jan	Cholera	60.5	25.1	5.6	22.6
	1993/Nov	Shigellosis	123.3	27.2	12.9	27.8
	1994/May–Jul	Cholera	191.3	28.0	15.5	29.6
	1995/Jun–Sep	Typhoid	298.7	27.6	18.4	29.1
	1996/Apr–Jun	Typhoid	159.0	27.6	12.1	28.2
Mekong River Delta	1993/Apr	Typhoid	37.0	29.2	5.7	28.2
	1993/Jun–Jul	Cholera	250.8	28.1	16.1	29.8
	1995/Jan–Mar	Typhoid	40.2	27.3	5.8	24.9
	1995/Apr–Jun	Cholera	161.38	28.5	11.9	29.4
	1995/Jun–Jul	Typhoid	280.7	28.4	15.9	30.3

PREC, precipitation (mm); TEMP, mean temperature (°C); VAP, vapor pressure (hPa); WET, wet days (number in month).
